# Genomic Characterization of *Aeromonas dhakensis* Isolated From a Fatal Dolphin Case

**DOI:** 10.1111/1758-2229.70410

**Published:** 2026-08-31

**Authors:** Kohei Yamazaki, Yu Takahashi, Yuki Yoneda, Takashige Kashimoto, Tsutomu Kakuda

**Affiliations:** ^1^ Laboratory of Veterinary Public Health, School of Veterinary Medicine Kitasato University Towada Japan; ^2^ Aomori Prefecture Central Livestock Hygiene Service Center Aomori Japan; ^3^ Laboratory of Animal Hygiene, School of Veterinary Medicine Kitasato University Towada Japan

**Keywords:** *Aeromonas dhakensis*, core genome phylogeny, dolphin, MLST, virulence genes, whole‐genome sequencing

## Abstract

*Aeromonas dhakensis* has emerged as a significant pathogen affecting both aquatic animals and humans; however, genomic data for isolates from marine mammals remain scarce. In this study, we characterised the genome of *A. dhakensis* strain KDL‐001, isolated from a fatal dolphin case, using whole‐genome sequencing and comparative genomics. Taxonomic analyses, including MLST and average nucleotide identity (ANI), confirmed the isolate as *A. dhakensis*. Core‐genome phylogeny further revealed that KDL‐001 is closely related to strains derived from fish and aquatic environments. Notably, in silico screening of virulence‐associated genes showed that the virulence‐associated gene profile of the dolphin isolate was broadly comparable to those of other *A. dhakensis* strains, with no isolate‐specific virulence‐associated genes being identified within the limits of this analysis. These findings demonstrate that the dolphin‐derived isolate is genomically comparable to previously described *A. dhakensis* strains and possesses conserved virulence‐associated genes commonly found within the species.

## Graphical abstract

Comparative genomic analysis of an *Aeromonas dhakensis* isolate recovered from a fatal dolphin case revealed close relatedness to previously described strains and a broadly comparable virulence‐associated gene profile, including *rtxA*, with no isolate‐specific virulence‐associated annotations identified.

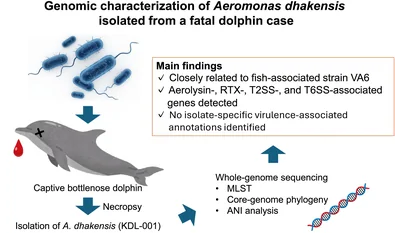

## 1 Introduction

*Aeromonas* species are ubiquitous Gram‐negative bacteria widely distributed in aquatic environments and are recognised as important pathogens of fish and other aquatic animals (Janda and Abbott 2010; Fernández‐Bravo and Figueras 2020; Parker and Shaw 2011; Beaz‐Hidalgo and Figueras 2013; Bartie and Desbois 2024). Amongst them, *Aeromonas dhakensis* has emerged as a clinically important species associated with severe and sometimes rapidly progressive infections in both humans and animals (Bartie and Desbois 2024; Rasmussen‐Ivey et al. 2016). Notably, a proportion of isolates historically reported as *Aeromonas hydrophila* in severe infections have been reassigned to *A. dhakensis* following advances in *Aeromonas* taxonomy and genome‐based identification, suggesting that the clinical impact of *A. dhakensis* may have been underrecognised (Bartie and Desbois 2024; Rasmussen‐Ivey et al. 2016). Consistent with this view, *A. dhakensis* is frequently described as carrying a broad repertoire of virulence‐associated genes, including aerolysin‐ and RTX‐associated genes and multiple secretion systems, which have been implicated in septicemia and acute fatal disease in susceptible hosts (Janda and Abbott 2010; Fernández‐Bravo and Figueras 2020; Suarez et al. 2012; Ponnusamy et al. 2014; Parker et al. 1994; Talagrand‐Reboul et al. 2020).

Although *Aeromonas* infections have occasionally been reported in dolphins, most cases were identified as *A. hydrophila* using conventional phenotypic methods (Cusick and Bullock 1973; Pérez et al. 2015). Consequently, genome‐based characterisation of *A. dhakensis* isolates recovered from dolphins remains unavailable. Whether dolphin‐derived isolates possess genomic features distinct from those of previously described *A. dhakensis* strains also remains unknown. Accurate identification of the causative agent and assessment of its pathogenic potential are therefore essential; however, closely related *Aeromonas* species are difficult to distinguish using conventional phenotypic methods alone (Fernández‐Bravo and Figueras 2020; Parker and Shaw 2011; Ajmi et al. 2025; Liu et al. 2022; Ghatak et al. 2016).

To address the diagnostic challenges associated with such cases, whole‐genome sequencing provides a robust framework for resolving taxonomic relationships and assessing genomic relatedness amongst *Aeromonas* species through approaches such as multilocus sequence typing (MLST) (Jolley et al. 2018), core‐genome phylogenetic analysis and average nucleotide identity (ANI) (Goris et al. 2007; Jain et al. 2018). In addition, comparative analysis of virulence‐associated gene repertoires allows evaluation of whether isolates associated with severe systemic infection possess distinctive virulence gene profiles.

In this study, we performed whole‐genome sequencing and comparative genomic analyses of an *A. dhakensis* KDL‐001 recovered from a dolphin that died suddenly. We aimed to determine the taxonomic and phylogenetic position of the dolphin‐derived isolate using MLST, core‐genome phylogeny and ANI analysis, and to compare it with publicly available *A. dhakensis* genomes.

## 2 Materials and Methods

### 2.1 Case Description

A 13‐year‐old female bottlenose dolphin ( *Tursiops truncatus* ) developed reduced appetite 1 day before death and was found dead the following morning with bloody discharge from the blowhole. A complete necropsy was performed on the day of death by veterinarians at the Aomori Prefectural Livestock Hygiene Service Center. Tissue samples were aseptically collected for bacteriological examination. Motile β‐hemolytic Gram‐negative bacilli were isolated from multiple organs, including the lung, heart, liver, kidney, spleen, and intestinal contents. Pulsed‐field gel electrophoresis confirmed that isolates recovered from different organs were genetically indistinguishable, and one representative isolate (KDL‐001) was used for whole‐genome sequencing. No similar clinical signs or mortality were observed in the remaining dolphins housed in the same facility. The isolate used in this study originated from a routine diagnostic investigation and no live animals were used for research purposes.

### 2.2 Genome Sequencing and Assembly

Genomic DNA was extracted from the dolphin‐derived *A. dhakensis* isolate KDL‐001 using standard procedures. Whole‐genome sequencing was performed on an Illumina iSeq 100 platform using 2 × 150 bp paired‐end reads. A total of 979,522 reads were generated. Raw reads were quality‐trimmed using CLC Genomics Workbench v25.0.3 (QIAGEN), and de novo assembly was performed using the default assembly settings in CLC Genomics Workbench v25.0.3. Assembly quality metrics, including total genome size, N50 value, GC content, and contig number, were assessed, and the resulting draft genome was used for downstream analyses. Genome annotation was performed using DFAST with default parameters (Tanizawa et al. 2018).

### 2.3 MLST Analysis

MLST was performed using the *Aeromonas* spp. scheme available in the PubMLST database (Jolley et al. 2018). Allele numbers for the six housekeeping genes (*gyrB*, *groL*, *gltA*, *metG*, *ppsA,* and *recA*) were assigned based on sequence similarity, and the corresponding sequence type (ST) was determined.

### 2.4 Core‐Genome Phylogenetic Analysis

Publicly available *A. dhakensis* genomes were selected from the NCBI RefSeq and GenBank databases. Genomes were included if they were annotated as *A. dhakensis*, publicly accessible and available as assembled genome sequences at the time of analysis. To capture the genomic diversity of the species, genomes originating from different hosts and environmental sources were included when available. Details of the strains and genome assemblies included in the comparative analysis are provided in Table S1.

Genome assemblies were annotated using Prokka to ensure consistent annotation across datasets (Seemann 2014). Core‐genome analysis was conducted using Panaroo (Tonkin‐Hill et al. 2020). Orthologous gene clusters were identified, and genes present in at least 95% of the genomes were defined as core genes. Amino acid sequences of core genes were aligned individually and concatenated to generate a core‐genome alignment. Columns with high entropy were removed using the default entropy‐based filtering implemented in Panaroo (Tonkin‐Hill et al. 2020). A maximum‐likelihood phylogenetic tree was inferred from the concatenated core‐genome alignment using IQ‐TREE v3.0.1 (Nguyen et al. 2015). The best‐fit substitution model was selected automatically using ModelFinder (Kalyaanamoorthy et al. 2017). Branch support was evaluated with 1000 ultrafast bootstrap replicates. The tree was rooted using *A. hydrophila* strains as the outgroup, and branch lengths represent the number of substitutions per site.

### 2.5 ANI Analysis

ANI analysis was performed to quantitatively assess genomic relatedness between the dolphin‐derived isolate KDL‐001 and reference *A. dhakensis* genomes (Goris et al. 2007; Jain et al. 2018). Genome assemblies in FASTA format were obtained from the NCBI RefSeq and GenBank databases and used without further modification. Pairwise ANI values were calculated using fastANI v1.34 with default parameters in an all‐versus‐all comparison framework (Jain et al. 2018). For downstream analysis and visualisation, ANI values between the dolphin‐derived isolate KDL‐001 and each *A. dhakensis* genome were extracted, and self‐comparisons were excluded.

### 2.6 Identification of Virulence‐Associated Genes

Virulence‐associated genes were identified using the virulence factor annotation function implemented in DFAST (Tanizawa et al. 2018), which incorporates reference information from the Virulence Factor Database (VFDB) (Chen et al. 2005). The analysis was performed using the default DFAST settings, with no additional user‐defined identity, coverage or *E*‐value thresholds. Genes assigned the same VFDB‐derived annotation were considered present, and annotations representing equivalent biological functions (e.g., aerolysin‐associated genes) were grouped for comparative analysis. DFAST annotation results were manually reviewed to confirm the presence of selected virulence‐associated genes in each genome. Comparative analyses were performed across all analysed *Aeromonas* genomes, including *A. dhakensis* and non‐dhakensis species.

## 3 Results

### 3.1 Genome Assembly and General Features

The draft genome of the dolphin‐derived *A. dhakensis* KDL‐001 consisted of 191 contigs, with a total length of 4,791,428 bp and a GC content of 61.9%. The assembly showed an N50 value of 59,865 bp and a gap ratio of 0.0076%. Genome annotation identified 4232 predicted coding sequences, 4 rRNA genes and 68 tRNA genes, with an overall coding ratio of 85.3%. In addition, one CRISPR locus was detected. MLST using the *Aeromonas* spp. scheme assigned the dolphin‐derived isolate KDL‐001 to sequence type ST‐2258 (Table 1). The allelic profile was *gyrB*‐1012, *groL*‐616, *gltA*‐1026, metG‐395, *ppsA*‐105, *recA*‐1103. The assigned MLST profile was consistent with classification as *A. dhakensis*.

**TABLE 1:** Multilocus sequence typing (MLST) profile of the dolphin‐derived *Aeromonas dhakensis* isolate.

Locus	Allele number
*gyrB*	1012
*groL*	616
*gltA*	1026
*metG*	395
*ppsA*	105
*recA*	1103
Sequence type (ST)	ST‐2258

*Note:* MLST was performed using the *Aeromonas* spp. scheme available in the PubMLST database.

### 3.2 Core‐Genome Phylogeny of the Dolphin‐Derived *A. dhakensis* KDL‐001

**FIGURE 1:**
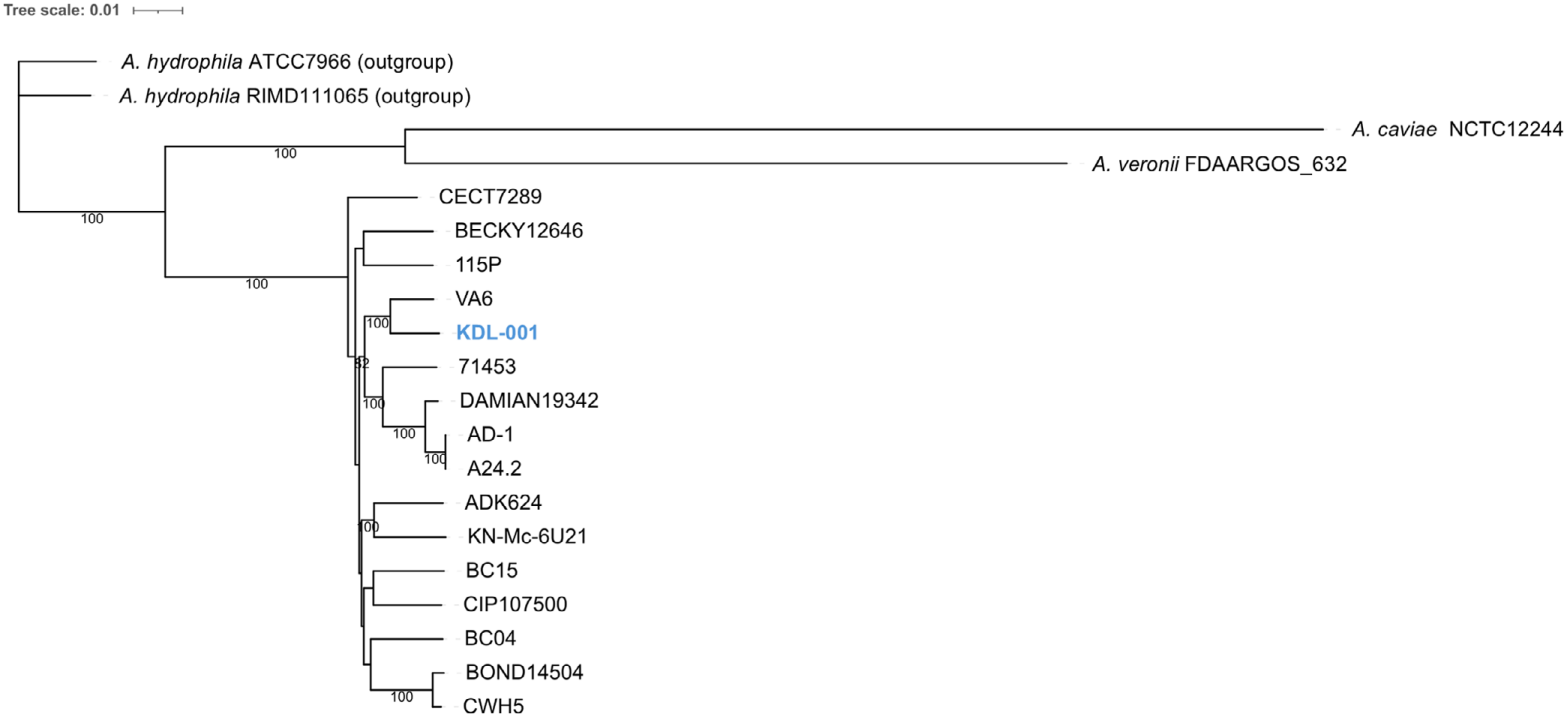
Core‐genome phylogeny of *Aeromonas* spp. The phylogenetic tree was constructed from a concatenated alignment of 277 core genes identified by Panaroo and inferred using the maximum‐likelihood method implemented in IQ‐TREE. *Aeromonas hydrophila* strains were used as the outgroup. Bootstrap support values greater than 70% are shown at the corresponding nodes. Branch lengths represent the number of substitutions per site.

To determine the phylogenetic position of the dolphin‐derived *Aeromonas* isolate, a core‐genome phylogenetic analysis was performed using representative *Aeromonas* genomes. The resulting maximum‐likelihood tree based on the concatenated alignment of core genes demonstrated that the dolphin‐derived isolate KDL‐001 clustered robustly within the *A. dhakensis* clade (Figure 1). This isolate was clearly separated from other *Aeromonas* species, including *A. hydrophila* , *A. caviae* and *A. veronii* , which formed distinct outgroup branches (Figure 1). Within the *A. dhakensis* cluster, the dolphin‐derived isolate KDL‐001 grouped closely with strains previously isolated from aquatic and fish‐associated sources, indicating a close evolutionary relationship.

### 3.3 ANI Analysis

**FIGURE 2:**
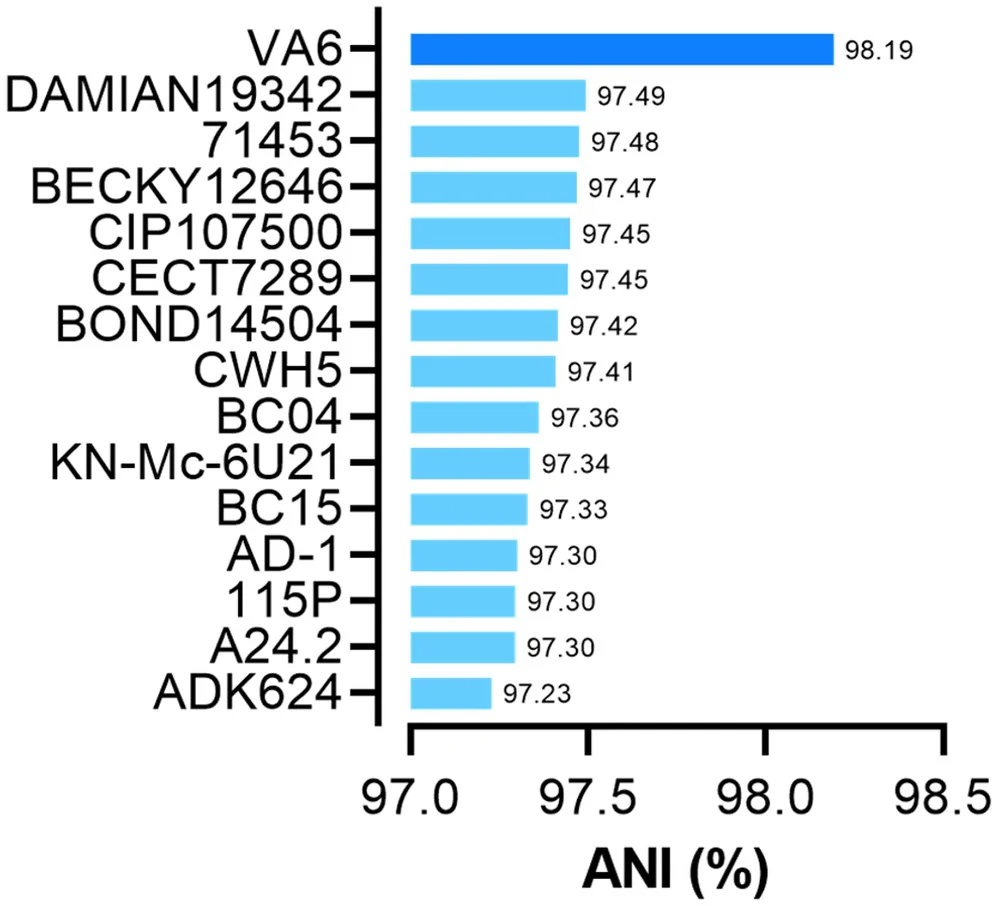
ANI comparison between the dolphin‐derived isolate KDL‐001 and *Aeromonas dhakensis* genomes. Average nucleotide identity (ANI) values were calculated using fastANI for pairwise comparisons between the dolphin‐derived isolate KDL‐001 and representative *A. dhakensis* genomes.

Pairwise ANI values were calculated amongst the analysed genomes to assess genomic relatedness. The dolphin‐derived isolate showed high ANI values (> 97%) with all *A. dhakensis* genomes included in this study (Figure 2). The highest ANI value was observed between the dolphin‐derived isolate and VA6, a strain originating from fish and aquatic environments. This pattern was consistent with the clustering observed in the core‐genome phylogeny. Overall, the ANI results indicate that the dolphin‐derived isolate is genomically closest to fish‐associated *A. dhakensis* strains within the dataset.

### 3.4 Virulence‐Associated Gene Profiles

**FIGURE 3:**
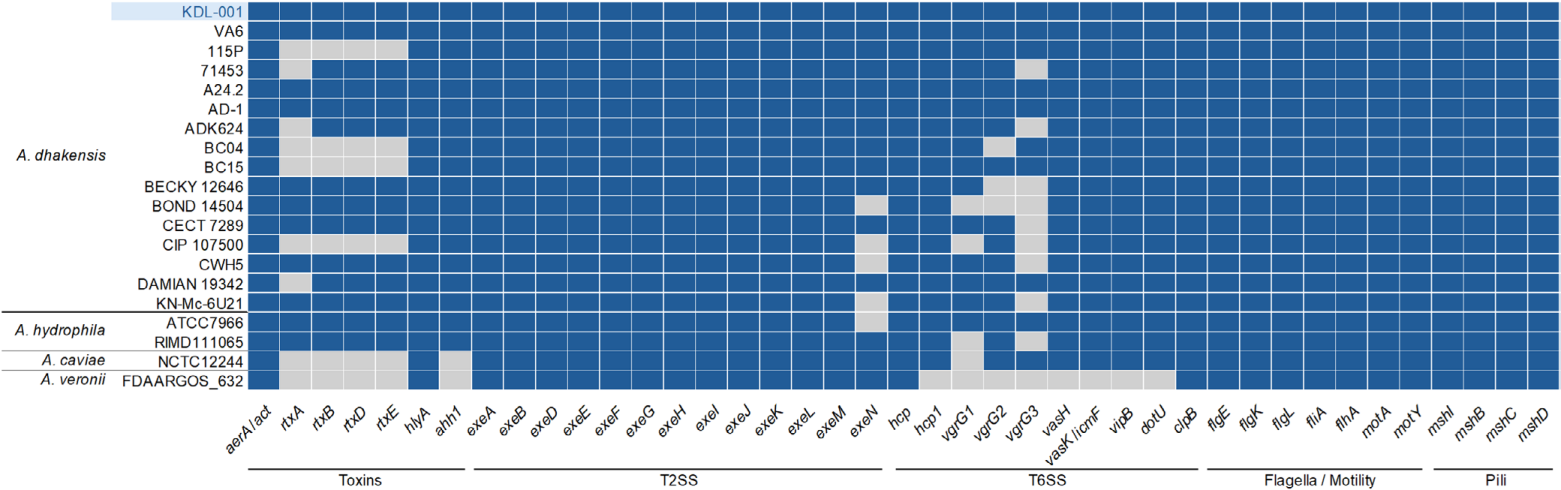
Presence and absence of virulence‐associated genes amongst Aeromonas strains. A heatmap showing the distribution of virulence‐associated genes identified by DFAST‐based virulence factor annotation across 20 *Aeromonas* strains. Rows represent strains ordered according to the core‐genome phylogeny, and columns represent virulence‐associated genes grouped by functional categories: Toxins, Type II secretion system, Type VI secretion system, flagella/motility and pili. Blue and grey cells indicate gene presence and absence, respectively. The dolphin‐derived isolate KDL‐001 is highlighted in blue.

The distribution of virulence‐associated genes across the analysed *Aeromonas* genomes was examined using presence/absence profiling based on DFAST annotations (Figure 3). Overall, the virulence‐associated gene repertoire was broadly comparable amongst *A. dhakensis* strains, including the dolphin‐derived isolate. Most *A. dhakensis* genomes harboured aerolysin‐associated genes (*aerA/act*) and genes within the RTX toxin locus (e.g., *rtxA*). RTX‐associated genes, including *rtxA*, were detected in KDL‐001 and were also identified in multiple *A. dhakensis* genomes. Genes encoding components of the Type II secretion system (*exe* genes) and the Type VI secretion system (*hcp* and *vgrG*) were also broadly conserved within the *A. dhakensis* group (Bartie and Desbois 2024). Notably, genes encoding the Type III secretion system, which are present in *A. veronii* , were not detected in the dolphin‐derived isolate or in other *A. dhakensis* strains analysed (Janda and Abbott 2010; Fernández‐Bravo and Figueras 2020; Beaz‐Hidalgo and Figueras 2013; Ponnusamy et al. 2014; Yamazaki et al. 2021; Gavín et al. 2002). Overall, the virulence gene repertoire of the dolphin‐derived isolate was comparable to that of other *A. dhakensis* strains, with no clearly isolate‐specific virulence‐associated annotation profile identified in this analysis.

## 4 Discussion

In this study, we characterised the dolphin‐derived *A. dhakensis* isolate KDL‐001 using whole‐genome sequencing and comparative genomic analyses. Genome assembly statistics and MLST analysis consistently classified the isolate as *A. dhakensis*, and both core‐genome phylogeny and ANI analysis demonstrated close relatedness to fish‐ and aquatic environment‐associated *A. dhakensis* strains (Table 1, Figures 1 and 2). These findings indicate that the dolphin‐derived isolate does not represent a distinct or host‐restricted lineage, but rather falls within the known genomic diversity of *A. dhakensis*.

The dolphin‐derived isolate showed high ANI values (> 97%) with all analysed *A. dhakensis* genomes and clustered within the *A. dhakensis* clade in the core‐genome phylogeny (Figures 1 and 2). Together, these results provide robust evidence supporting classification of the isolate as *A. dhakensis* (Janda and Abbott 2010; Fernández‐Bravo and Figueras 2020; Seemann 2014; Tonkin‐Hill et al. 2020). Notably, the closest genomic relative was strain VA6, which originated from fish and aquatic environments. This finding is consistent with the broad distribution of *A. dhakensis* and related *Aeromonas* species in aquatic hosts and environments (Fernández‐Bravo and Figueras 2020; Beaz‐Hidalgo and Figueras 2013; Bartie and Desbois 2024). However, although the closest genomic relative was a fish‐associated strain, the source of infection could not be determined in the present study.

Analysis of virulence‐associated genes revealed that the annotation profile of the dolphin‐derived isolate was broadly comparable to those of other *A. dhakensis* strains (Figure 3). KDL‐001 harboured aerolysin‐associated genes, RTX‐associated genes and components of the Type II and Type VI secretion systems. These findings indicate that the isolate possesses virulence‐associated factors commonly reported in *Aeromonas* spp. (Janda and Abbott 2010; Fernández‐Bravo and Figueras 2020; Beaz‐Hidalgo and Figueras 2013; Bartie and Desbois 2024; Suarez et al. 2012; Ponnusamy et al. 2014; Parker et al. 1994). RTX toxins have been implicated in cytotoxicity and tissue damage in *Aeromonas* spp. (Suarez et al. 2012; Ponnusamy et al. 2014). Although the contribution of *rtxA* to the clinical outcome in this dolphin cannot be determined from the present genomic analysis alone, its potential role warrants further investigation. Future studies combining comparative genomics with experimental infection models will be necessary to clarify the contribution of RTX toxins to systemic infection. Because the present analysis was based on a draft genome assembly, interpretation of gene absence should be made with caution, as assembly fragmentation may affect the recovery and annotation of some genomic regions.

Most publicly available *A. dhakensis* genomes used for comparative analysis originated from aquatic or fish‐associated sources, with limited and unevenly reported metadata regarding geographic origin and host. Although many of the genomes analysed in this study were isolated in Southeast Asia, particularly Vietnam, broadly similar phylogenetic relationships and virulence‐associated annotation profiles were observed amongst the strains included in the present dataset.

The present study is limited by the analysis of a single isolate recovered from a single dolphin case. Therefore, conclusions regarding host adaptation, transmission routes or the broader epidemiology of *A. dhakensis* in marine mammals cannot be drawn from the current dataset. In addition, other potential infectious, toxicological and non‐infectious causes of death were not systematically investigated and therefore cannot be excluded.

Taken together, our results indicate that the dolphin‐derived *A. dhakensis* isolate is genomically comparable to previously described strains isolated from aquatic and fish‐associated sources. These findings underscore the value of genome‐based approaches for accurate identification and comparative characterization of *Aeromonas* isolates. However, the findings should be interpreted within the limitations of a single‐case genomic investigation. From a veterinary and microbiological perspective, they support continued awareness of *A. dhakensis* as a potential opportunistic pathogen in aquatic animals.

## Author Contributions

**Kohei Yamazaki:** conceptualization, data curation, formal analysis, funding acquisition, investigation, methodology, software, visualization, writing – original draft. **Takashige Kashimoto:** supervision, writing – review and editing. **Tsutomu Kakuda:** project administration, validation, resources, writing – review and editing. **Yuki Yoneda:** data curation, resources. **Yu Takahashi:** data curation, resources.

## Funding

This work was supported by Japan Society for the Promotion of Science (22K14998, 22KK0289).

## Ethics Statement

No experimental procedures involving live animals were performed in this study. The isolate analysed originated from a routine diagnostic investigation.

## Conflicts of Interest

The authors declare no conflicts of interest.

## Supporting information


**Table S1:** Metadata and assembly accession numbers of *Aeromonas dhakensis* genomes included in the comparative genomic analysis.

## Data Availability

The draft genome sequence of the dolphin‐derived *Aeromonas dhakensis* isolate has been deposited in the DDBJ/ENA/GenBank database under accession numbers BAAJYU010000001–BAAJYU010000190 (BioProject PRJDB35631, BioSample SAMD01816750). All genome assemblies used for comparative analyses are publicly available from the NCBI RefSeq and GenBank databases.
