# Supplementary material for: Genomic Characterization of Aeromonas dhakensis Isolated From a Fatal Dolphin Case
Source: Environ Microbiol Rep. 2026 Aug 31;18(5):e70410. doi: 10.1111/1758-2229.70410 (PMC13529358; doi:10.1111/1758-2229.70410)
Supplement: Supplementary file 1 [file EMI4-18-e70410-s001.pdf]

Supplementary Table 1. Metadata and assembly accession numbers of Aeromonas dhakensis genomes included in the comparative genomic analysis

**Strain**	**Species**	**Source/Host**	**Geographic location**	**Assembly Accession**
KDL-001	*A. dhakensis*	Bottlenose dolphin (*Tursiops truncatus*)	Japan	BAAJYU010000001–BAAJYU010000190
VA6	*A. dhakensis*	Fish (*Mastacembelus* sp.)	Ho Chi Minh City, Vietnam	GCA_037101205.1
DAMIAN 19342	*A. dhakensis*	Clinical isolate	NA	GCA_029773105.1
KN-Mc-6U21	*A. dhakensis*	Nutria (*Myocastor coypus*)	Korea	GCA_002285935.1
CIP 107500	*A. dhakensis*	NA	NA	GCA_000820305.1
CWH5	*A. dhakensis*	Fish (*Ictalurus punctatus*)	Zhuhai, Guangdong, China	GCA_043099375.1
CECT 7289	*A. dhakensis*	NA	NA	GCA_000819705.1
BOND 14504	*A. dhakensis*	Clinical isolate	NA	GCA_029773285.1
BECKY 12646	*A. dhakensis*	Clinical isolate	NA	GCA_051106055.1
BC15	*A. dhakensis*	River water	Ghana	GCA_026651465.1
115P	*A. dhakensis*	Fish (*Carassius auratus*)	Bursa, Turkey	GCA_051920185.1
71453	*A. dhakensis*	Human clinical isolate	Ningbo, China	GCA_020405305.1
A24.2	*A. dhakensis*	One Health enteric sample	Vietnam	GCA_054343875.1
AD-1	*A. dhakensis*	Fish (*Pangasianodon hypophthalmus*)	Vietnam	GCA_052059445.1
ADK624	*A. dhakensis*	NA	NA	GCA_963425745.1
BC04	*A. dhakensis*	River water	Ghana	GCA_026651365.1

NA indicates that host or geographic information was not reported in the corresponding public genome record or associated publication.
